# Supplementary material for: Human microcephaly protein RTTN interacts with STIL and is required to build full-length centrioles
Source: Nat Commun. 2017 Aug 15;8:247. doi: 10.1038/s41467-017-00305-0 (PMC5558016; doi:10.1038/s41467-017-00305-0)
Supplement: Supplementary file 1 — Supplementary Information [file 41467_2017_305_MOESM1_ESM.pdf]

File Name: Supplementary Information
Description: Supplementary Figures and Supplementary Table

File Name: Supplementary Movie 1
Description: Live-cell imaging of RPE1-based *RTTN*$^{-/-}$; *p53*$^{-/-}$ cell expressing GFP-CP110 and mCherry-H2B (related to Supplementary Figure 4a).

File Name: Supplementary Movie 2
Description: Live-cell imaging of RPE1-based *RTTN*$^{-/-}$; *p53*$^{-/-}$ cell expressing GFP-CP110 and mCherry-STIL (related to Supplementary Figure 4b).

File Name: Peer Review File
Description:

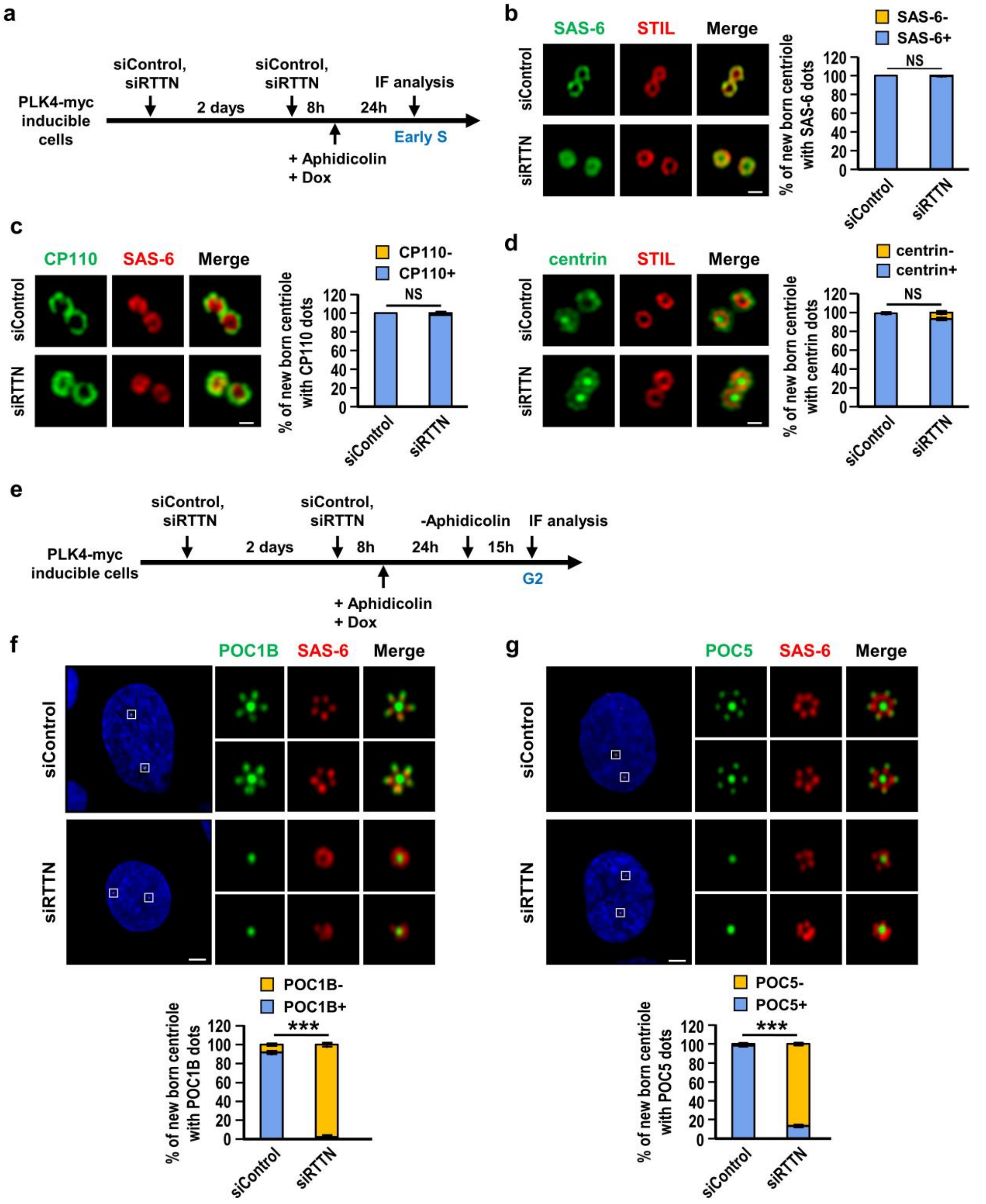

Supplementary Figure 1. Depletion of RTTN has no effect on initial procentriole assembly but it does inhibit the recruitment of POC5 and POC1B to distal-half centrioles. (**a-d**) PLK4-myc inducible cells were treated with siControl or siRTTN as shown in **a**, and analyzed by confocal fluorescence microscopy using antibodies against SAS-6/STIL (**b**), CP110/SAS-6 (**c**), and centrin/STIL (**d**). Histogram illustrating the percentages of newborn centrioles with SAS-6 (**b**), CP110 (**c**), or centrin (**d**) dots. Error bars represent the mean ± s.d. (n=3 independent experiments with 100 cells scored per experiment). (**e-g**) PLK4-myc-inducible cells were treated with siControl or

siRTTN as shown in **e**, and analyzed by confocal fluorescence microscopy using antibodies against POC1B/SAS-6 (**f**) and POC5/SAS-6 (**g**). Histograms illustrate the percentages of newborn centrioles with POC1B (**f**) or POC5 (**g**) dots. Error bars represent the mean ± s.d. (n=3 independent experiments with 100 cells scored per experiment). ****P*<0.001; NS, not significant (two-tailed *t*-test). Scale bar, 0.5 μm in **b-d**; Scale bar, 5 μm in **f** and **g**.

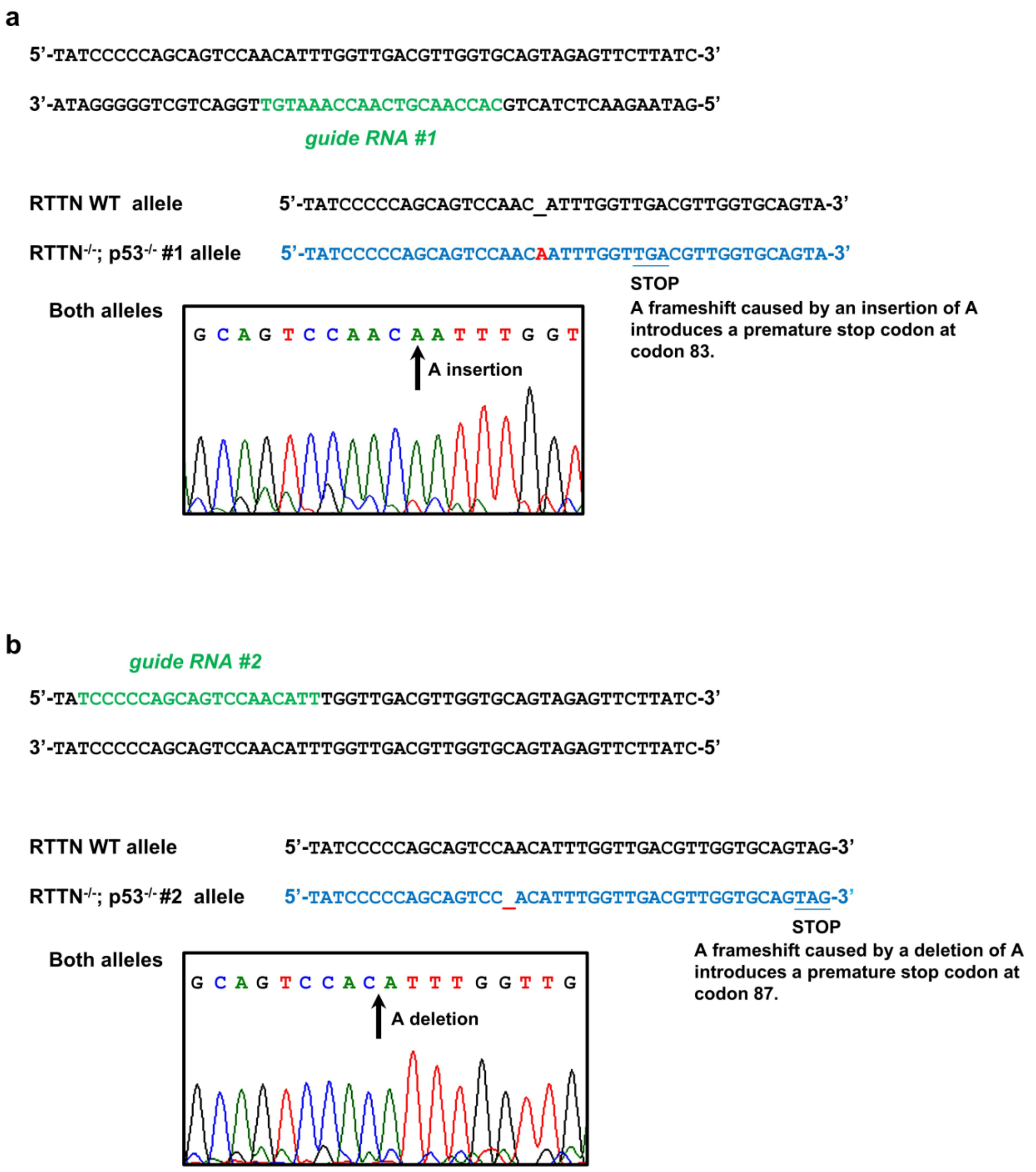

Supplementary Figure 2. Analysis of *RTTN*$^{-/-}$*; p53*$^{-/-}$ RPE1 cell lines (#1 and #2). (**a**,**b**) Sequence analysis of the *RTTN* gene in genomic DNA isolated from *RTTN*$^{-/-}$*; p53*$^{-/-}$ RPE1 cell lines #1 (**a**) and #2 (**b**). Both wild-type (black) and mutant alleles (blue) are shown. The location of the mutation (deletion or insertion) is marked in red.

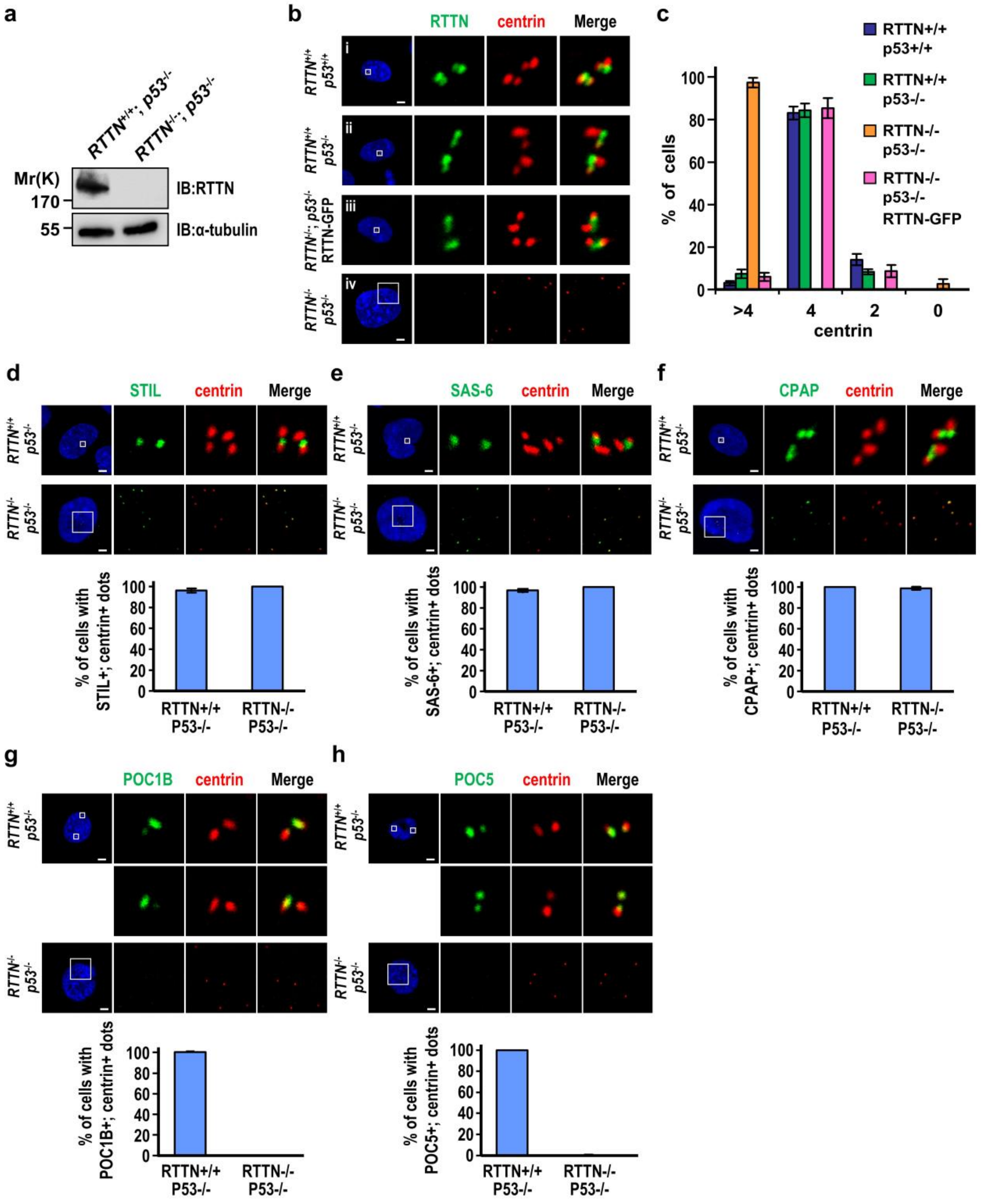

Supplementary Figure 3. Loss of RTTN in *RTTN*$^{-/-}$*; p53*$^{-/-}$ RPE1 cells induces *de novo* amplification of PPBs that lack POC1B and POC5. (**a**) RPE1-based *RTTN*$^{+/+}$*; p53*$^{-/-}$ or *RTTN*$^{-/-}$*; p53*$^{-/-}$ cells were analyzed by immunoblotting using the indicated antibodies. (**b**) RPE1-based cells derived from *RTTN*$^{+/+}$*; p53*$^{+/+}$*, RTTN*$^{+/+}$*; p53*$^{-/-}$, *RTTN*$^{-/-}$*; p53*$^{-/-}$, or *RTTN*$^{-/-}$*; p53*$^{-/-}$ cells expressing wild type RTTN-GFP were synchronized at early S phase by aphidicolin treatment for 24 h, immunostained with the indicated antibodies, and analyzed by confocal microscopy. (**c**) Histogram illustrating the percentages of cells exhibiting centrin signals. Error bars represent the mean ± s.d.

from three independent experiments (n=100/experiment). (**d-f**) *RTTN*$^{+/+}$*; p53*$^{-/-}$ or *RTTN*$^{-/-}$*; p53*$^{-/-}$ RPE1 cells were synchronized at early S phase and stained with the indicated antibodies as described in **b**. Histograms illustrate the percentages of cells exhibiting STIL (**d**), SAS-6 (**e**) or CPAP (**f**) signals. (**g,h**) *RTTN*$^{+/+}$*; p53*$^{-/-}$ or *RTTN*$^{-/-}$*; p53*$^{-/-}$ RPE1 cells were synchronized with aphidicholin for 24 h, and then released in fresh medium for another 15 h to allow progression to G2 phase. The cells were fixed and stained with the indicated antibodies. Histograms illustrate the percentages of cells exhibiting POC1B (**g**) or POC5 (**h**) signals. **(d-h)** Error bars represent the mean ± s.d. from three independent experiments (n=100/experiment). Scale bar, 5 µm. $^{+/+}$ indicates wild type gene, $^{-/-}$ indicates mutant gene.

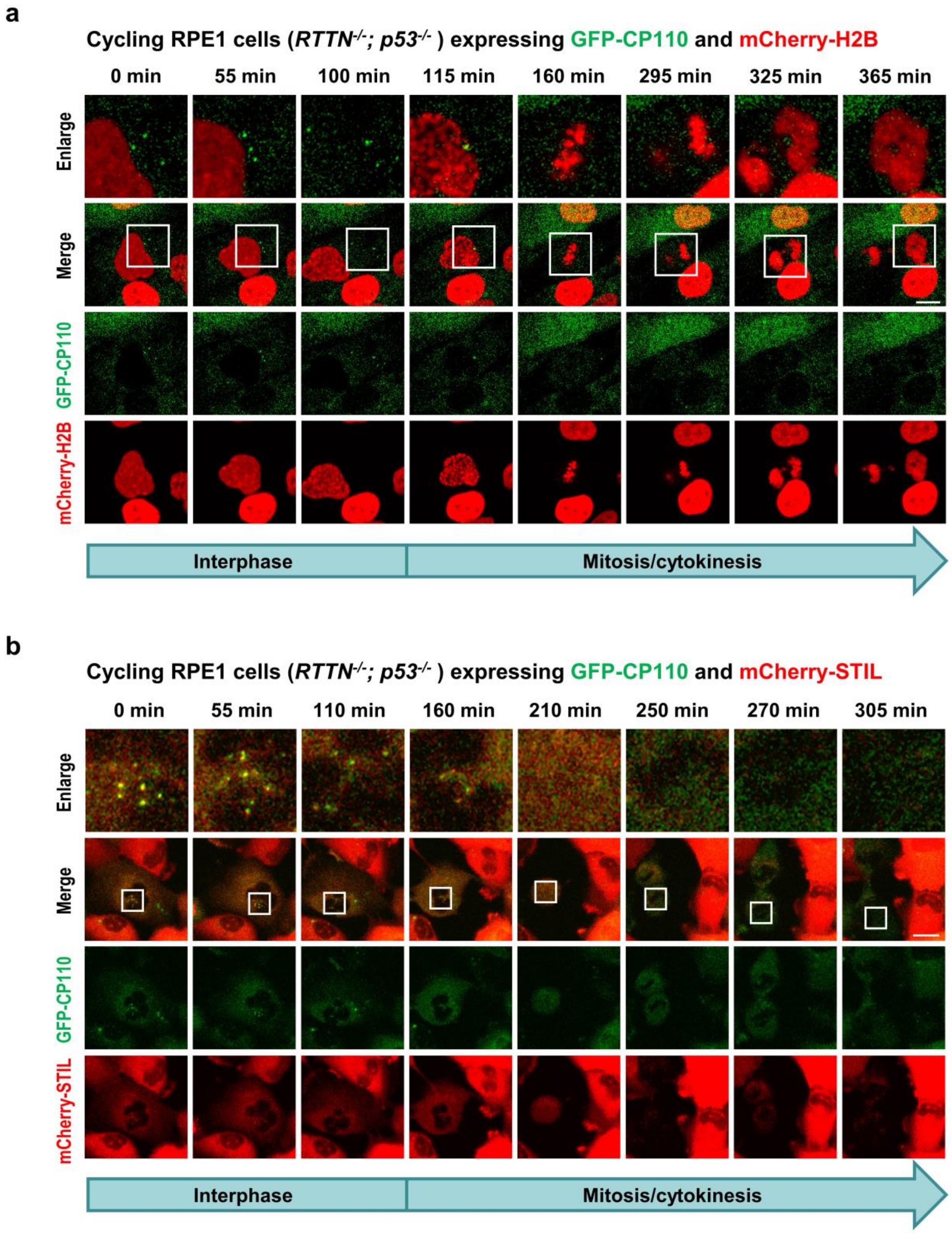

Supplementary Figure 4. Live-cell imaging of the assembly of *de novo* PPBs during the cell cycle. (**a**) Time-series images of RPE1-based *RTTN*$^{-/-}$*; p53*$^{-/-}$ cells expressing GFP-CP110 (green) and mCherry-H2B (red). (**b**) Time-series images of RPE1-based *RTTN*$^{-/-}$*; p53*$^{-/-}$ cells expressing GFP-CP110 (green) and mCherry-STIL (red). The live-cell images were taken at the indicated time points (minutes) using an LSM780 Carl Zeiss confocal system. Scale bar, 20 µm. See also Supplementary Movies 1 and 2.

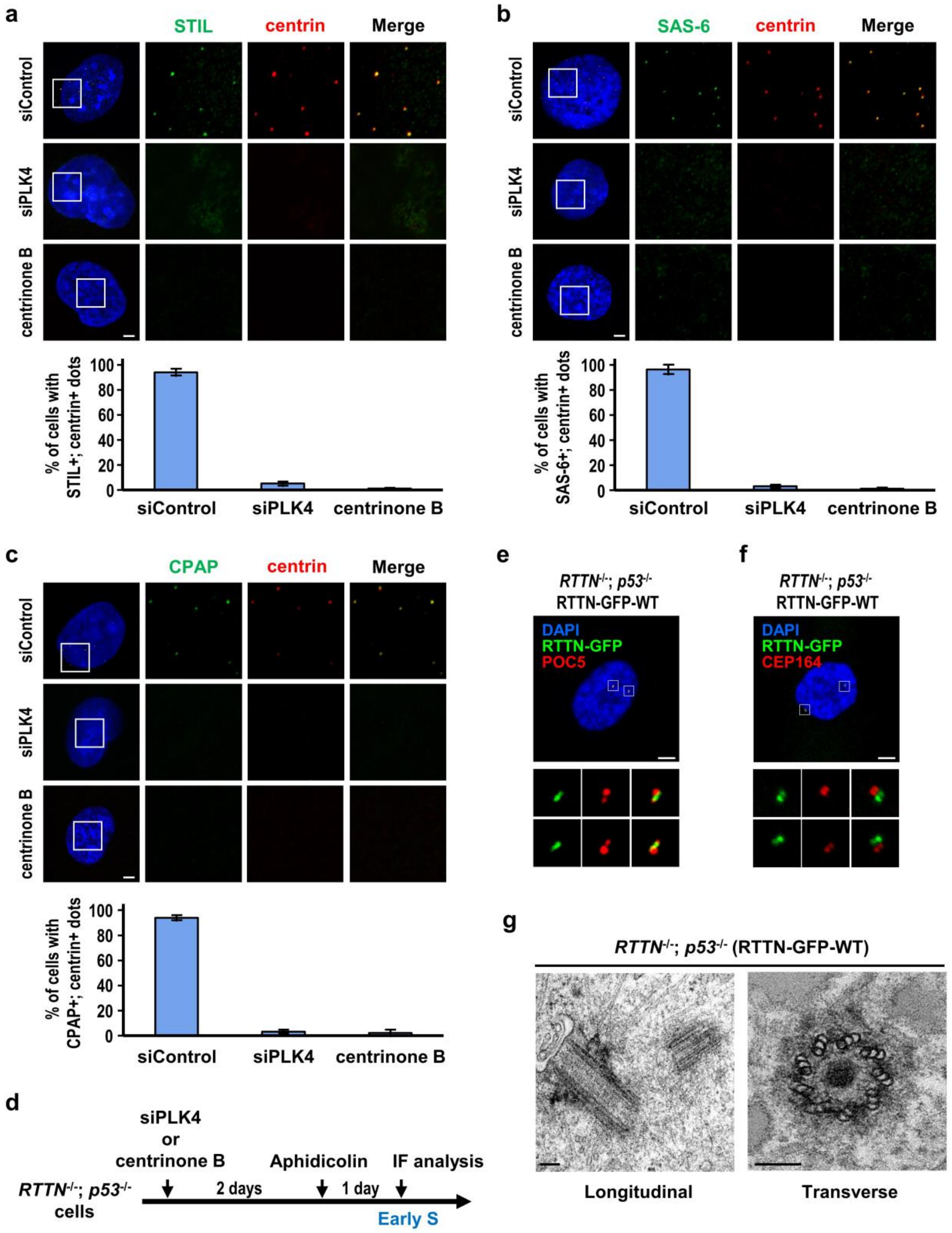

Supplementary Figure 5. The generation of PPBs in *RTTN*$^{-/-}$*; p53*$^{-/-}$ RPE1 cells was inhibited in siPLK4- or centrinone B-treated cells and rescue experiments. (**a-c**) *RTTN*$^{-/-}$*; p53*$^{-/-}$ RPE1 cells were transfected with siPLK4 or treated with centrinone B (a PLK4 inhibitor, 300 nM) as shown in **d**, and analyzed by confocal fluorescence microscopy using the indicated antibodies. Histograms illustrate the percentages of cells exhibiting STIL/centrin (**a**), SAS-6/centrin (**b**), CPAP/centrin (**c**) signals. Error bars represent the mean ± s.d. from three independent experiments (n=100/experiment).

(**e-g**) Rescue experiments. (**e,f**) *RTTN*$^{-/-}$*; p53*$^{-/-}$ cells expressing wild-type RTTN (RTTN-GFP-WT) were immunostained with the indicated antibodies and analyzed by confocal microscopy. (**g**) *RTTN*$^{-/-}$*; p53*$^{-/-}$ cells expressing RTTN-GFP-WT were analyzed by electron microscopy. Scale bar, 5 µm in **a-c**, **e** and **f**; Scale bar, 100 nm in **g**.

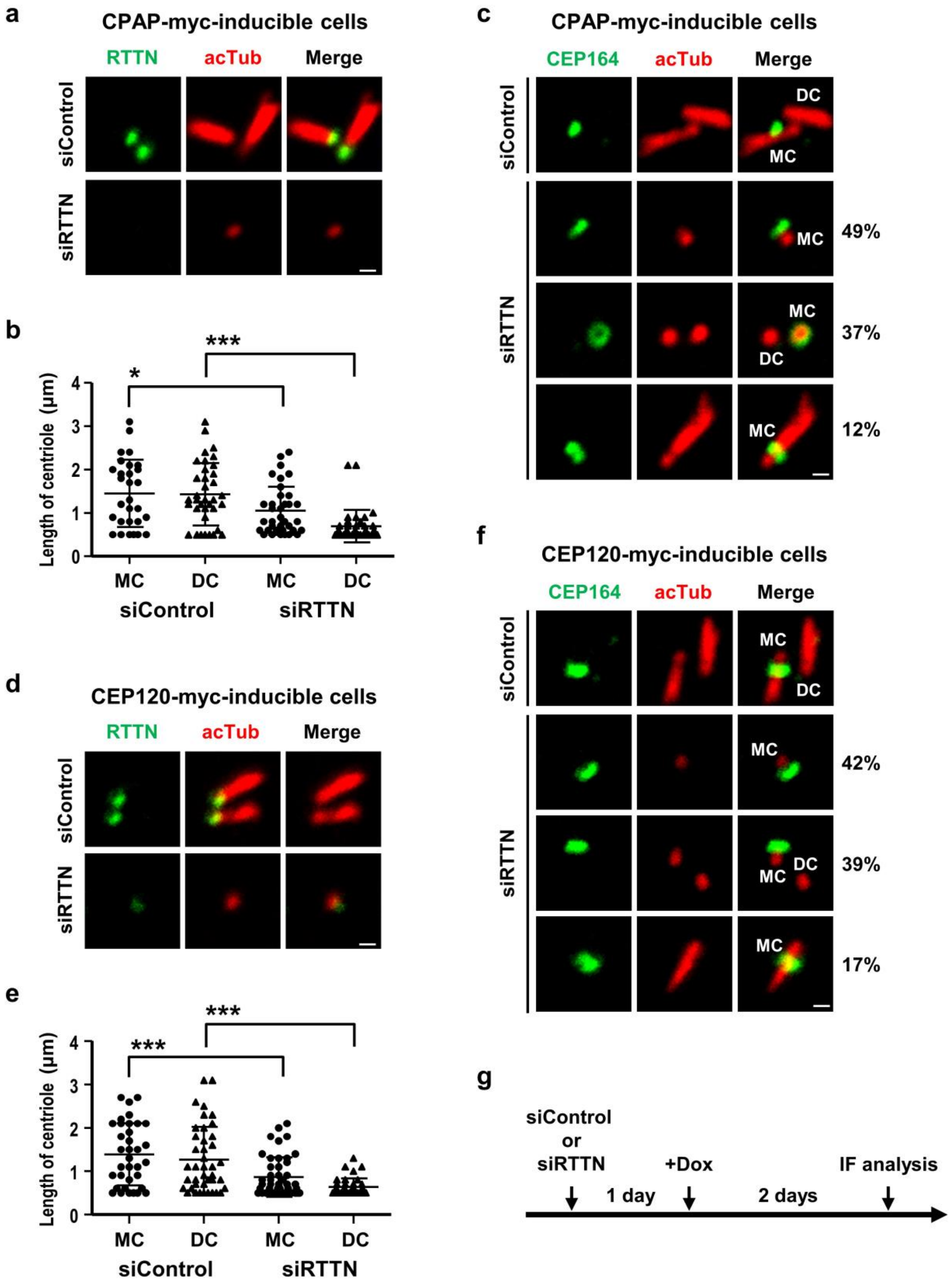

Supplementary Figure 6. Depletion of RTTN inhibits the CPAP- or CEP120-induced overly long centrioles (> 0.5 μm). CPAP-myc- (**a-c**) or CEP120-myc- (**d-f**) inducible cells were treated as shown in **g.** The cells were fixed and immunostained with antibodies against RTTN, acTub, or CEP164 (a distal appendage protein that labels the mother centriole). Histograms illustrate the length of mother centrioles (MC) or daughter centrioles (DC) in CPAP-myc- (**b**) or CEP120-myc-inducible cells (**e**). Error

bars represent the mean ± s.d. *P<0.05; ***P<0.001 (two-tailed *t*-test). Scale bar, 0.5 µm.

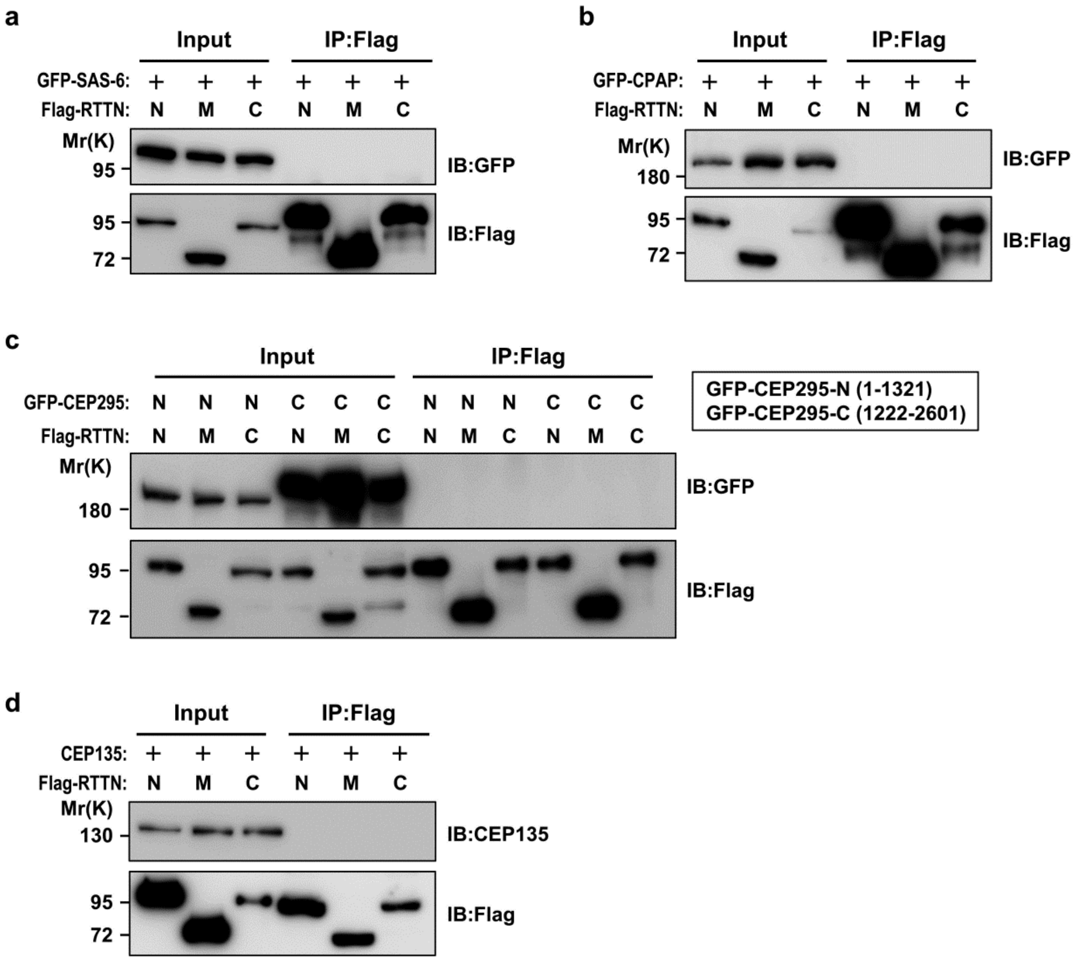

Supplementary Figure 7. Testing for interactions between RTTN and other centriolar proteins. (**a-c**) HEK 293T cells were co-transfected with vectors encoding various Flag-RTTN-truncation constructs plus GFP-SAS-6 (**a**), GFP-CPAP (**b**), or GFP-CEP295 fragments (**c**). Twenty-four hours after transfection, cell lysates were immunoprecipitated (IP) with anti-Flag and then immunoblotted (IB) using the indicated antibodies. (**d**) HEK 293T cells were transfected with vectors encoding various Flag-RTTN-truncation constructs. Twenty-four hours after transfection, cell lysates were IP with anti-Flag antibody and IB using antibodies against CEP135 or Flag.

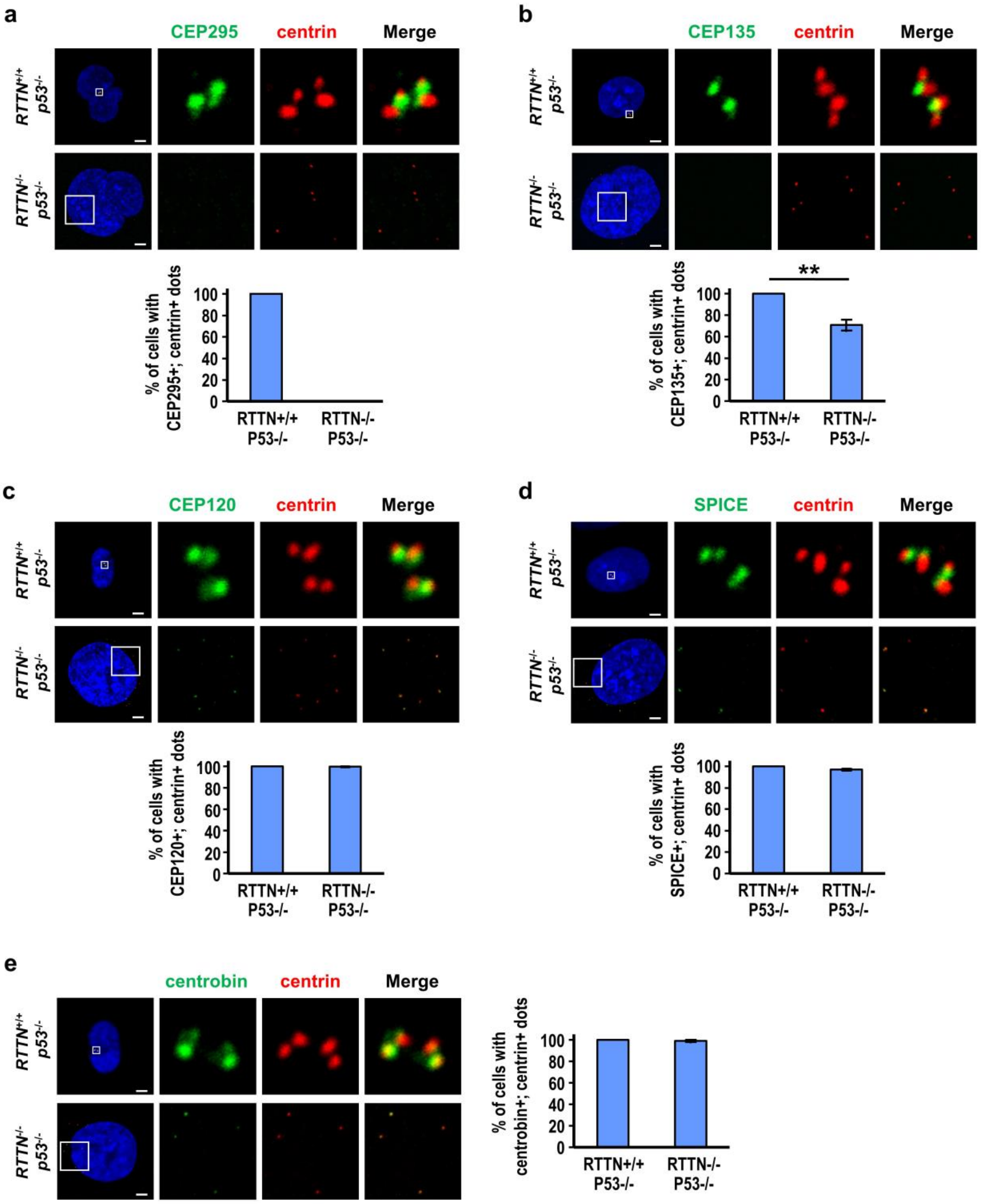

Supplementary Figure 8. Loss of RTTN produces PPBs that lack CEP295. (**a-e**) *RTTN*$^{+/+}$*; p53*$^{-/-}$ or *RTTN*$^{-/-}$*; p53*$^{-/-}$ RPE1 cells were synchronized at early S phase by aphidicolin treatment for 24 h, immunostained with the indicated antibodies and analyzed by confocal microscopy. Histograms illustrate the percentages of cells exhibiting CEP295 (**a**), CEP135 (**b**), CEP120 (**c**), SPICE (**d**), or centrobin (**e**) signals. Error bars represent the mean ± s.d. from three independent experiments (n=100/experiment). $**P<0.01$ (two-tailed t-test). Scale bar, 5 µm.

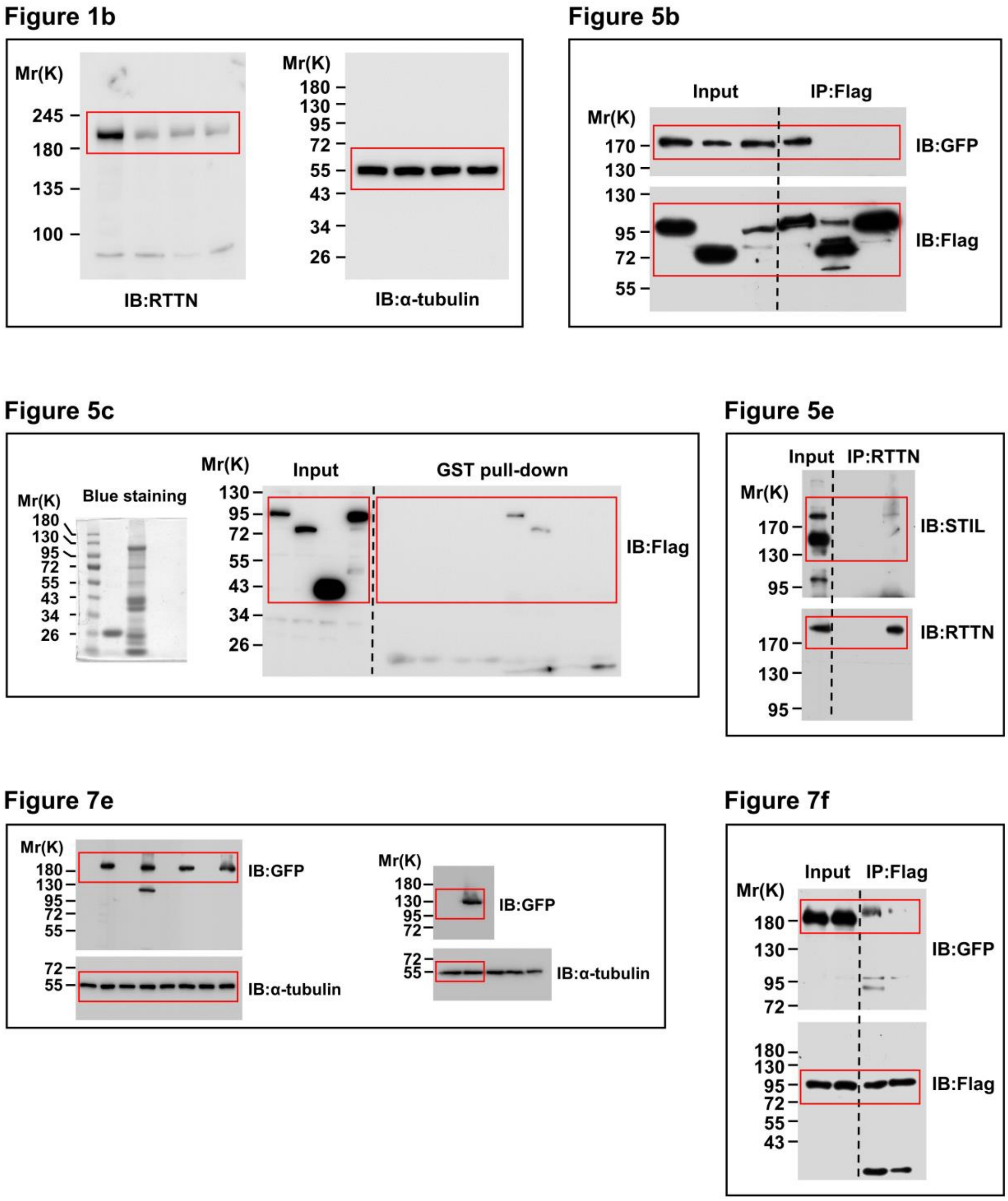

Supplementary Figure 9. Uncropped images of Western blots shown in the main text.

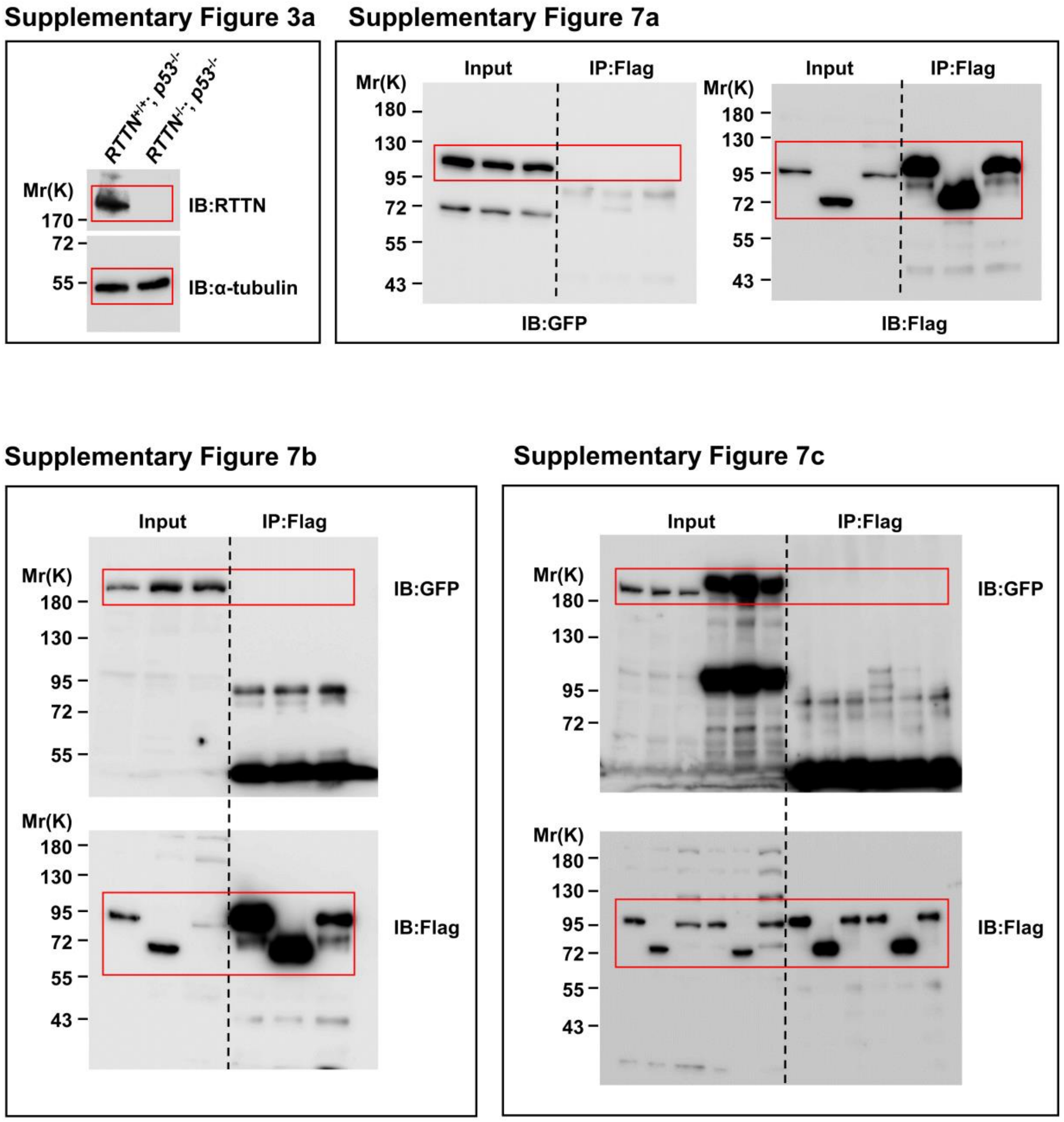

Supplementary Figure 10. Uncropped images of Western blots shown in Supplementary Figure 3a and Supplementary Figure 7a-c.

Supplementary Figure 7d

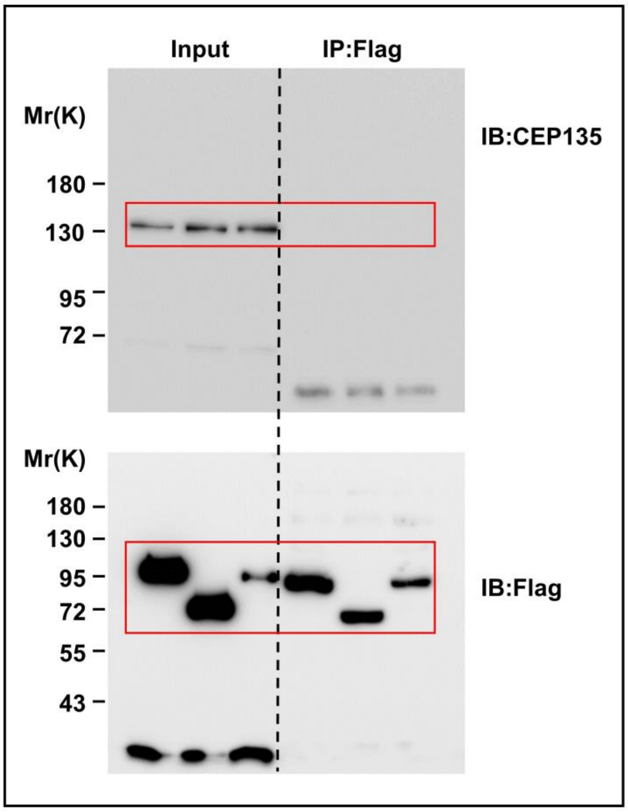

Supplementary Figure 11. Uncropped images of Western blots shown in Supplementary Figure 7d.

Supplementary Table 1: siRNA sequences

Name	Sequence 5'-3'	
PLK4	GACACUGACACAGUCAAGAACACAU	(ref. 8)
STIL	AUUUCCAGCAGAAACUGUUUGGAGC	(ref. 8)
CPAP	AGAAUUAGCUCGAAUAGAAUU	(ref. 13)
CEP135	UUUACAAGGAGUUCAUCACUCAGUC	(ref. 9)
CEP120	AAUAUAUCUUCUUGCAUCUCCUUCC	(ref. 15)
CEP295	GACUGUUAGUGAAAUUGAGAGUAAA	(ref. 19)
SPICE	UCUAAACUCUCAGUCUAACACGAAU	(ref. 19)
centrobin	CGAAGCUUGUCAGTCGGAUUGGAAA	(ref. 19)
siRTTN#1	CCCGUGUCUUCACUUUGCAAUGGAA	this study
siRTTN#2	CCUUUGGGCUCUGAUUUACAAUUAU	this study
siRTTN#3	CAUGCUCAUGUAAACUCCAGGAUAU	this study
